# A simple technique to classify diffraction data from dynamic proteins according to individual polymorphs

**DOI:** 10.1107/S2059798321013425

**Published:** 2022-02-18

**Authors:** Thu Nguyen, Kim L. Phan, Dima Kozakov, Sandra B. Gabelli, Dale F. Kreitler, Lawrence C. Andrews, Jean Jakoncic, Robert M. Sweet, Alexei S. Soares, Herbert J. Bernstein

**Affiliations:** a Department of Computer Science, Stony Brook University, Stony Brook, NY 11794-2424, USA; b Department of Medicine, Oncology, Biophysics and Biophysical Chemistry, Johns Hopkins University, 725 North Wolfe Street, Baltimore, MD 21205, USA; c Department of Applied Mathematics and Statistics, Stony Brook University, Stony Brook, NY 11794-3600, USA; d National Synchrotron Light Source II, Building 745, Brookhaven National Laboratory, PO Box 5000, Upton, NY 11973-5000, USA; e Ronin Institute for Independent Scholarship, c/o NSLS-II, Building 745, Brookhaven National Laboratory, PO Box 5000, Upton, NY 11973-5000, USA

**Keywords:** chymotrypsinogen, clustering, polymorphs, protein dynamics, unit-cell changes

## Abstract

One often observes small but measurable differences in the diffraction data measured from different crystals of a single protein. These differences might reflect structural differences in the protein and may reveal the natural dynamism of the molecule in solution. Partitioning these mixed-state data into single-state clusters is a critical step that could extract information about the dynamic behavior of proteins from hundreds or thousands of single-crystal data sets. Mixed-state data can be obtained deliberately (through intentional perturbation) or inadvertently (while attempting to measure highly redundant single-crystal data). To the extent that different states adopt different molecular structures, one expects to observe differences in the crystals; each of the polystates will create a polymorph of the crystals. After mixed-state diffraction data have been measured, deliberately or inadvertently, the challenge is to sort the data into clusters that may represent relevant biological polystates. Here, this problem is addressed using a simple multi-factor clustering approach that classifies each data set using independent observables, thereby assigning each data set to the correct location in conformational space. This procedure is illustrated using two independent observables, unit-cell parameters and intensities, to cluster mixed-state data from chymotrypsinogen (ChTg) crystals. It is observed that the data populate an arc of the reaction trajectory as ChTg is converted into chymotrypsin.

## Abstract 2

The dynamics of proteins can be explored from polymorphs observed by the clustering of multiple data wedges.

## 1. Introduction

Proteins often undergo structural changes as part of their normal functioning. Crystal structures often reveal proteins in different conformations (called polymorphs). Crystallography explores an average structure of all of the molecules in the volume that the X-ray beam interrogates: an immense number of individual molecules with possibly significantly different polymorphs. These different structures might have revealed information on the dynamics of transitions among those polymorphs had they not all been averaged together. Because of this averaging, instead of seeing distinct states clearly, we may see only what looks like blurred thermal motion.

To reduce this problem, a typical structural study of a protein might involve constraining the molecule in one of its states by binding a ligand before crystal growth. For example, in the emerging field of biological data storage, proteins with two distinct conformations (called polystates) are intentionally switched between them to represent binary code (0 and 1; Sethi, 2015 ▸). It seems likely that molecules in a single crystal form may show slightly different structures depending on the pH, the state of hydration *etc*. That is, they exhibit dynamic behavior that may or may not indicate changes related to their function. Here, we will adopt the term ‘polystates’ to refer to protein structural polymorphs that correspond to biologically relevant conformations of proteins, as well as to other significant state variations. To observe them, one might sample these variables carefully to find states where all molecules are the same in a crystal. We aim in this work to design a more general workflow.

Modern crystallographic practice provides opportunities to discover and analyze the sort of changes that we describe here: we use crystals small enough that each may contain only one polystate. In particular, data collection at a synchrotron source often includes the measurement of many partial sets of crystal diffraction data from many, often very small, crystals (Liu *et al.*, 2011 ▸; Giordano *et al.*, 2012 ▸; Rossmann, 2014 ▸; Assmann *et al.*, 2016 ▸; Bernstein *et al.*, 2017 ▸, 2020 ▸; Gao *et al.*, 2018 ▸). This is possible because fourth-generation synchrotron sources are very bright, the X-ray beams are very small, the 2D detectors employed are very fast and modern goniometers are very precise. With a detector operating at 200 Hz, a 360° rotational sweep with 0.2° per image will take approximately 9 s. These are standard experimental parameters at the FMX and AMX beamlines at National Synchrotron Light Source II (NSLS-II), where a beam size even smaller than the small crystals is employed. We could sample hundreds of tiny crystals (1–5 µm), each perhaps one polymorph, and then separate them into these polymorphs.

For this sort of treatment, one may mount crystals singly or, say, with half a dozen in each sample mount (loop or mesh micro-mount); in each case, data are obtained for each individual crystal. The crystals may come from different crystallization drops, or even different preparations, but they are all nominally isomorphous; our objective is to improve the quality of the data by merging multiple measurements. In practice one may partition the data from many crystals into different clusters, based on differences in intensities or unit-cell parameters. Each cluster may represent one step in the normal dynamic motion of the molecule. Starting from multiple independent samples increases the chances of having multiple states to observe.

As we mentioned, two popular criteria for clustering data sets are the similarity in unit-cell parameter values and in reflection intensities. Unit-cell databases have long been used for substance identification and are now used as a coarse screen for molecular-replacement candidates. Steno (1669 ▸), as cited in Authier (2013 ▸), noticed the constancy of interfacial angles of crystals. Reflection intensities represent the true structure, so similarity of reflections is a good metric to use in comparing data sets.

We demonstrate our approach using chymotrypsinogen (ChTg). ChTg is the precursor (zymogen) of the mammalian digestive enzyme chymotrypsin (CHT), one of several well known serine proteases (Kunitz & Northrop, 1935 ▸; Siekevitz & Palade, 1960 ▸). This conversion is accomplished by several enzymatic cleavages. Firstly (in the digestive tract), trypsin cleaves the peptide bond between Arg15 and Ile16 to yield π-CHT, which is an active enzyme form. Secondly, π-CHT molecules autolyze one another to cleave the bonds between Leu13 and Ser14 to release Ser14-Arg15, and between Tyr146 and Thr147 and between Asn148 and Ala149 to release Thr147-Asn148. The resulting α-chymotrypsin is formed by three chains tethered by disulfide bridges.

We measured 146 diffraction data sets, each from a single crystal of ChTg crystallized in three different conditions: pH 4.6, pH 5.6 and pH 6.5. We discovered that the polymorphs that we observed resemble the partial conversion of ChTg to CHT. Almost all of the crystals that we successfully assembled into clusters, from which we could average data and solve the structure, were formed at pH 6.5. To detect different molecular states in individual crystals, we based the partitioning of the single-crystal data on the following two properties: unit-cell parameters and intensities.

We first employed differences in unit-cell parameters as a conventional method for clustering single-crystal data. However, this was clearly inadequate, and we extended this to partitioning on similarities in diffraction intensities. The criterion for similarity was the correlation coefficient calculated between pairs of measurements, thereby classifying according to the slightly different but stable conformational states that generated these data. We found that clustering by correlation of intensities also revealed the large unit-cell differences that were observed. In general, however, unit-cell differences are observable much earlier during structure determination than distinctive intensity differences, and they can provide preliminary clustering with the use of correlation coefficients to follow.

## 2. Methods

### 2.1. Crystallization

**Figure 1:**
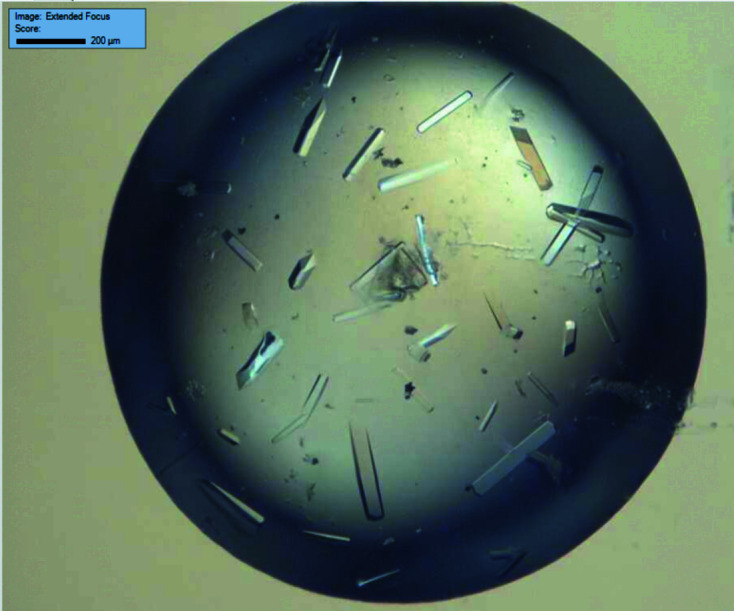
Representative ChTg crystals from crystallization conditions consisting of 1.0–2.0 *M* ammonium sulfate, 0.1 *M* MES pH 6.5, 10% or 15% dioxane.

We determined crystallization conditions for chymotrypsinogen (Sigma) using the commercial Crystal Screen HT (Hampton Research) set up with a Mosquito robot (STP Labtech). Crystals were grown via hanging-drop vapor diffusion at 18°C from condition F11. To optimize the crystallization conditions further, we set up a 24-well tray using hanging-drop vapor diffusion with a fixed pH of 6.5, varying the concentration of both dioxane (10% or 15%) and ammonium sulfate (1.0–2.0 *M*). Each drop, consisting of 1 µl reservoir solution (1.0–2.0 *M* ammonium sulfate, 0.1 *M* MES pH 6.5 and 10% or 15% dioxane) and 1 µl 10.0 mg ml^−1^ enzyme, was equilibrated over 0.5 ml reservoir solution. The other two crystallization conditions consisted of either 0.2 *M* ammonium acetate, 0.1 *M* sodium acetate trihydrate pH 4.6, 30%(*w*/*v*) PEG 4000 or 0.5 *M* ammonium sulfate, 0.1 *M* sodium citrate tribasic dihydrate pH 5.6, with 1.0 *M* lithium sulfate monohydrate in the reservoir. All crystals were cryocooled in 3.5 *M* lithium sulfate. A typical drop containing these crystals is shown in Fig. 1 ▸.

### 2.2. Data collection and structure-solution strategies

We used ChTg crystals of ∼50 µm in size and an ∼5 µm beam to obtain several hundred data sets at an energy of 13.48 keV (0.92 Å), collecting 120° of data per crystal on beamline 17-ID-1 (AMX) at NSLS-II, Brookhaven National Laboratory using a Dectris EIGER X 9M detector. For each crystal with sufficient data, the data set was indexed, integrated and scaled using a version of the data-reduction pipeline *fast_dp* (Winter & McAuley, 2011 ▸) modified to run in the local distributed computing environment, which supplies the following modules: *XDS* (Kabsch, 2010 ▸), *DIALS* (Winter *et al.*, 2018 ▸), *Phenix* (Afonine *et al.*, 2012 ▸), *AIMLESS* and *POINTLESS* (Evans & Murshudov, 2013 ▸). The tetragonal crystals of ChTg diffracted to between 2.0 and 2.4 Å resolution.

**Figure 2:**
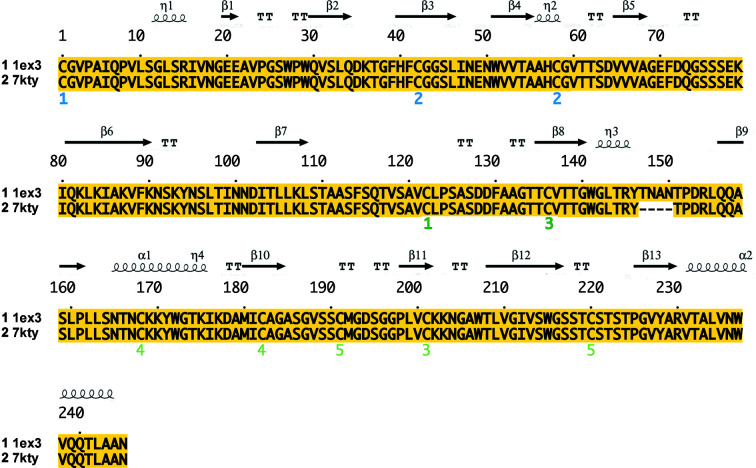
Sequence alignment of ChTg (PDB entry 1ex3) and the average structure (PDB entry 7kty). The loop residues 147–150 do not display electron density in PDB entry 7kty.

**Figure 3:**
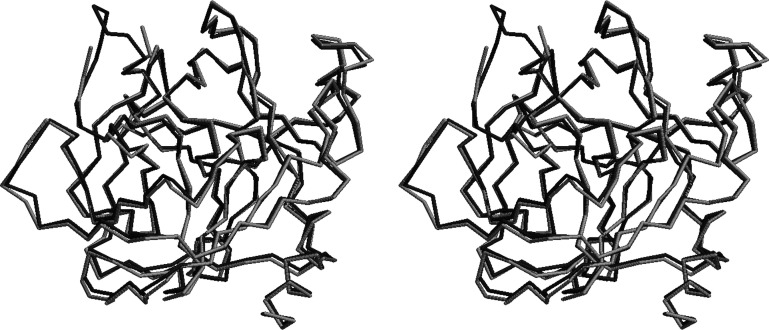
Cross-eyed stereoview of the structural alignment of PDB entry 1ex3 (dark gray) and the average structure PDB entry 7kty (light gray). The *FATCAT* chain r.m.s.d. is 0.56 Å. The regions with significant differences are adjacent and appear at the upper left of this figure. Firstly, in the average structure the amino acids between Thr147 and Asn150 are missing. Secondly, in the average structure the amino acids between Thr139 and Tyr146 adopt a significantly different conformation.

We determined the structure by molecular replacement with *Phaser* (McCoy *et al.*, 2007 ▸) using the structure of ChTg with PDB code 1ex3 as a model (Bernstein *et al.*, 1977 ▸; Berman *et al.*, 2000 ▸; Pjura *et al.*, 2000 ▸). The data were refined to their final resolution using iterative rounds of refinement with *REFMAC* (Murshudov *et al.*, 1997 ▸, 2011 ▸) and manual rebuilding in *Coot* (Emsley *et al.*, 2010 ▸). We then used this model to build our ‘average’ structure from an average of all data sets at an energy of 13.48 keV. Our average structure was broadly similar to previously published structures, with the exception of one loop that was not resolvable in our data (Thr147–Asn150; see Fig. 2 ▸) and one adjacent region that adopts a different conformation (Thr139–Tyr146; see Fig. 3 ▸). While scaling these data sets, we noted clusters in the data. It is possible that they originated from polymorphs in the ChTg crystals, which may represent dynamic behavior in the molecules.

### 2.3. Data-clustering program

We used a custom-modified version of the *KAMO* clustering pipeline (Yamashita *et al.*, 2018 ▸), which uses the clustering program *Blend* (Foadi *et al.*, 2013 ▸) to generate a dendrogram of the data sets. We expanded *KAMO* and *Blend* to allow two-factor clustering as follows. Unit-cell parameters and amplitudes contain independent information. One expects differences in unit-cell parameters to reflect changes in the outer shape of the structure, perhaps responding to the presence of internal or external ligands. On the other hand, differences in amplitudes will be sensitive to all conformational changes in the protein. Therefore, in a single workflow the new scheme obtains initial ‘coarse’ clusters according to the similarity of the crystallo­graphic unit cells (space-group clusters) and then generates ‘fine’ clusters by a further partitioning of each cluster according to the similarity of the amplitude data. (The modified software is available at https://github.com/nsls-ii-mx/blend and http://github.com/nsls-ii-mx/yamtbx.)

Our procedure employs Pearson correlation coefficient (CC) calculations to determine similarity scores, requiring that pairs of data sets have many measured amplitudes in common: one must have a reasonably complete set of structure factors. For this CC clustering, 70% completeness is required. One can introduce a penalty for unmatched structure factors, and can obtain a solution with a completeness as low as 20–40% (Bernstein *et al.*, 2017 ▸); we are studying the effect of even lower completeness to apply this procedure to partial data sets. We will show that this clustering approach demonstrates how increasingly sensitive clustering methods can identify increasingly detailed structural differences (Figs. 3 ▸–8).

In our study, when we ran *KAMO.multi_merge* with *Blend*, it reduced the data in different point groups (the observed point groups were exemplified by space groups *P*2, *P*222 and *P*422). We selected the data that reduced in *P*422 because these data contained the largest number of data sets and correspond to the published space group for ChTg; we aimed to use the structure factors to display the differences in the structures corresponding to the data that reduced in *P*422. We used *KAMO* to divide the data sets of the chosen space group into different clusters based on intensity CC. Please note that the term *space-group clustering* is in common usage, but the technically correct term for clustering performed prior to refinement is *point-group clustering*; for example, our algorithms clustered the cymotrypsinogen data using the exemplar space group *P*422 (No. 89) with its Laue point group (4/*m* 2/*m* 2/*m*), rather than the actual space group *P*4_1_2_1_2 (No. 92) adopted by all crystals.

For the clustering step, we defined the ‘distance’ between pairs of data sets as *d*(*i*, *j*) = [1 − CC(*i*, *j*)]^1/2^. Our procedure then used a hierarchical clustering analysis (Rokach & Maimon, 2005 ▸) with Ward clustering (Ward, 1963 ▸) to find distinct groups of the chosen data sets. In Ward clustering, the data sets are considered first by building a small cluster out of the two closest data sets and then adding one data set at a time to whichever data set or existing cluster results in a new cluster of smallest variance (mean of squares of distances). There are many other choices of what is called ‘linkage’ in forming a cluster dendrogram, such as using cluster centroids. Using the minimal variance allows the use of one simple distance matrix as input to the clustering algorithm, rather than requiring repeated calculation of distances among cells or, worse, among *hkl* vectors of structure factors, but it does tend to produce dendrograms for which the heights grow rapidly. Strauss & von Maltitz (2017 ▸) discuss some alternative linkage choices. The program outputs a dendrogram that illustrates the distances (differences) among clusters by the *y* axis (the joint variance). To obtain a certain number of clusters which contain more similar data sets, we chose a height-cutoff value *k* accordingly. The lower the *k* value, the more similar the data sets in each cluster are. Each cluster now relates to a structure built after merging data sets within it.

**Figure 4:**
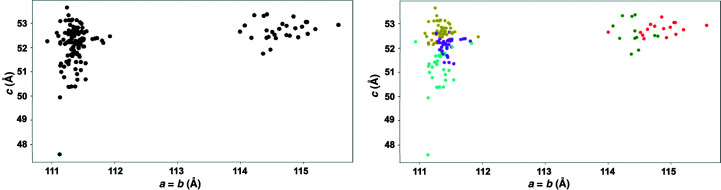
Two main data clusters can be identified by inspection (the *a* = 111 Å group and the *a* = 114–115 Å group). We observed that our data partitioned cleanly between 28 data sets with an *a* (= *b*) unit-cell parameter of approximately 114–115 Å and 118 data sets with an *a* (= *b*) unit-cell parameter of approximately 111 Å. The separation into the two unit-cell clusters is shown in the monochrome clustering on the left. The further division of these two clusters into amplitude-based clusters is shown by the colors on the right. The *a* = 114–115 Å unit-cell-based cluster contains the green and red clusters and the *a* = 111 Å unit-cell-based cluster contains the cyan, purple and yellow clusters. Each of our data sets was sufficiently large that amplitude-based clustering could have been used from the start. However, many serial crystallography projects consist of narrow wedges of data, each of which might be too small to cluster effectively using amplitudes because amplitude-based clustering requires that data sets have a sufficient number of observations in common. This figure illustrates how an initial use of cell-based clustering might be used to bootstrap amplitude-based clustering.

We want to understand how the unit-cell parameter values of data sets relate to the clusters determined by similarity in diffraction intensities. Since the space group is *P*4_1_2_1_2 (No. 89/92), the unit-cell parameters *a* and *b* are equal and all unit-cell angles are 90°. Since there are only two free parameters, we could visually demonstrate how the intensity clusters relate to the unit-cell parameters *a* and *c* for each data set (Fig. 4 ▸).

**Figure 5:**
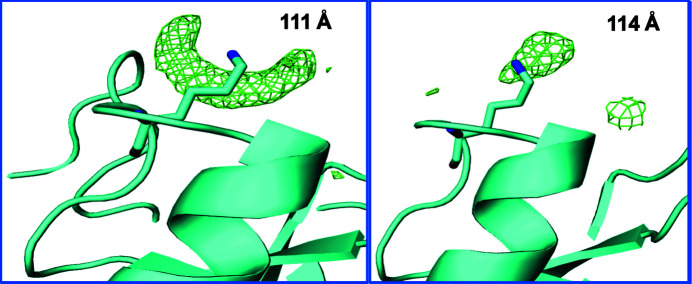
Differences in solvent between the *a* = 111 Å cluster and the *a* = 114–115 Å cluster. *F*_o_ − *F*_c_ electron difference density displaying two differences that we observed in solvent density between the *a *= 111 Å cluster and the *a* = 114–115 Å cluster (difference densities at 2σ are shown in green for both data sets). Left: ribbon diagram of ChTg around Lys175 (cyan) for the *a* = 111 Å cluster. Right: ribbon diagram of ChTg around Lys175 (cyan) for the *a* = 114–115 Å cluster. This density was modeled as a water molecule.

Finally, we created a molecular structure from the average intensities from all of the crystals in each intensity cluster and also averaged structures for each of the different unit-cell parameter clusters (see Fig. 5 ▸; the data are available from the corresponding authors). To create structures that relate to each of these clusters, we employed the average structure defined above in the data-collection section as the starting model for structure determination and refinement of the structures of each of the clusters. All of the processes that we used to build the structures of the clusters and to subsequently refine them are automated with the help of *REFMAC* (Murshudov *et al.*, 1997 ▸, 2011 ▸). Following the automated refinement steps, we performed a manual check-and-refine step using *Coot* (Emsley *et al.*, 2010 ▸) to ensure that no serious errors remained from the automated process and corrected the refined model as needed. *FATCAT* (Ye & Godzik, 2004 ▸) allowed us to quantify the morphological differences among structure solutions.

### 2.4. Illustrating the differences to identify physically meaningful clusters

Any software that uses observable parameters to generate clusters may generate a very large number of clusters. How is one to determine which clusters are physically meaningful? Dendrograms can illustrate the relationships among clusters, but one must illustrate physical relevance using structural tools, *i.e.* comparing the structures obtained from each of these clusters. We generated two software tools for this purpose (see https://github.com/nsls-ii-mx/chymotrypsinogen). Both tools use individual colors to differentiate among clusters, which we can then test for physical relevance, and both tools use two- or three-dimensional plots to illustrate an underlying physical characteristic of the structure.

We developed a tool to create color-coded coordinate ellipses. We plotted the *xyz* coordinates of the C^α^ atom of a particular amino acid in the structure that we observed to be highly mobile among the clusters. We created color-coded ellipsoids that enclosed all C^α^ atoms found from each of the individual clusters. The size of each ellipsoid indicates the variation of the coordinates within the corresponding cluster. Ideally, the size of each color-coded ellipsoid will not be very large compared with the separations among the centroids of the ellipsoids, indicating that each cluster represents a separable state. The code is available in the https://github.com/nsls-ii-mx/chymotrypsinogen git repository as the file https://raw.githubusercontent.com/nsls-ii-mx/chymotrypsinogen/master/ellipsoid.py. An example of the use of this graphic appears in Fig. 8.

**Figure 6:**
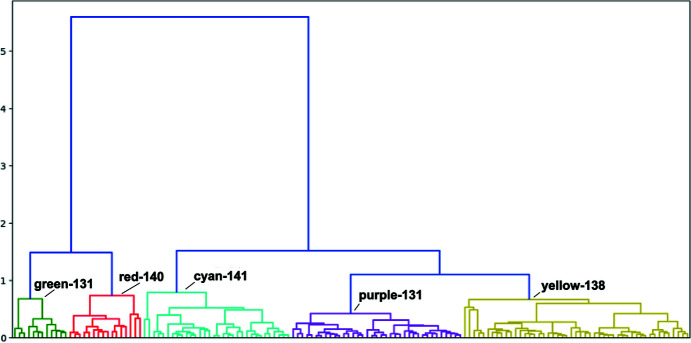
Amplitude-based clusters generated using *KAMO* (dendrogram). This dendrogram shows a representation of the similarity of pairs of data sets and of clusters of more data sets. They are arranged with the most similar clusters near each other and the connecting bar at a height corresponding to the distance between clusters. The difference was calculated using Ward’s method for hierarchical clustering, which yields a composite metric that contains information from amplitude differences and from unit-cell differences. Our algorithm is described in Section 2.3. Structures were solved corresponding to each of these 145 clusters. We deposited the structure derived from refinement against structure factors, each of which was an average of that observation from all of these clusters, as PDB entry 7kty. Through inspection of the derived structures we selected a height within the dendrogram at which to partition our data, giving five clusters. We then averaged all structure factors within each of the five distinct clusters and then refined against these to give cluster-average structures. We deposited the averaged structure from the green clusters as PDB entry 7ku1, from the red clusters as PDB entry 7ku2, from the cyan clusters as PDB entry 7ku3, from the purple clusters as PDB entry 7ktz and from the yellow clusters as PDB entry 7ku0.

We also plotted the *a* and *c* axis lengths for each data set that resides within an amplitude-based cluster (Fig. 4 ▸). Employing a dendrogram-plotting graphic tool from *KAMO*, we illustrated all data that originated from each postulated cluster in a different color (Fig. 6 ▸).

To detect subtle differences among the structures of the clusters, we used *FTMap* (Kozakov *et al.*, 2015 ▸), software that was designed to determine and characterize ligand-binding hotspots on the surfaces of proteins. The algorithm uses a library of 16 molecules as probes to discover potential patches on the surface of a structure where a molecule might bind. Differences in proposed surface binding could reveal otherwise unnoticeable physical differences among the structures.

## 3. Results

### 3.1. Data collection and protein structures

We collected 511 complete data sets and processed 325 of them using our data-reduction pipeline* fast_dp_nsls*2. Of these, 175 files had a resolution better than 4 Å. Finally, 146 data sets from the point group represented by space group No. 89 (*P*422) were merged using *Blend* cell-based cluster analysis. The protein is a single chain of 245 residues, of which four residues (147–150) were not resolved.

We obtained our initial structure, PDB entry 7kty, from a merge of all 146 data sets and called this the average structure (denoted thus in Tables 1 ▸ and 2 ▸). We used *REFMAC* and *Coot* to refine the structure and reduce the *R* value to about 18%.

Averaging all 146 data sets together resulted in a relatively high *R*_merge_ value (48%), but nevertheless PDB entry 7kty was a good fit to these data (*R*_work_ = 19%, *R*_free_ = 20%). This average structure is slightly different from the published structure with PDB code 1ex3 which we used as an initial phasing model. For example, PDB entry 7kty has a missing loop from residue 147 to residue 150, which is a characteristic of mature α-chymotrypsin. Fig. 2 ▸ displays the sequence alignment between PDB entry 1ex3 and our structure, PDB entry 7kty, with the elements of the secondary structure drawn on top.

### 3.2. Clustering with unit cells and with amplitudes

Clustering software will generate data corresponding to candidate polystates, even in cases where truly distinct polystates are not actually present in the samples. Two independent data sets collected from two samples will always give different average structures. Such differences are often not relevant in terms of dynamics or states when the differences are small compared with the experimental error. The only way to determine whether candidate clusters may correspond to biologically relevant polystates is to generate and examine the corresponding structural models (typically atomic models) with appropriate real-space tools, such as *FATCAT* and *Coot*. In the case of the ChTg data, we could see from inspection that the data could be divided into two large clusters corresponding to structures with *a* ≃ 111 Å and those with *a* ≃ 114–115 Å (Fig. 4 ▸).

Employing only the observed diffraction intensities, we identified two main clusters that corresponded to the two main polymorphs that ChTg adopted in our crystals, based on the length of the *a* axis. In addition, there were five clusters that corresponded to biologically relevant polymorphs present in our data. The cell-based clustering shows that the unit-cell lengths separate clearly into two groups, while the *c* unit-cell length varies less and is not clearly separable. There were significant solvent-region differences between the *a* = 111 Å cluster and the *a* = 114–115 Å cluster (Fig. 4 ▸).

When comparing the structures corresponding to the *a* = 111 Å cluster and the *a* = 114–115 Å cluster, we observed that the *a* = 114–115 Å cluster data yields observable density for all 245 residues (similar to PDB entry 1ex3), while the *a* = 111 Å cluster data indicate that there is a missing loop from residue 147 to residue 150 (this region is also not observed in the average structure). Another thing that we observed is the presence of strong density near Lys175 in the *a* = 111 Å cluster data, while the *a* = 114–115 Å cluster data does not have this large artifact (Fig. 5 ▸). We discuss these differences further below.

Using *Blend* and *KAMO*, we obtained 145 clusters from the 146 ChTg data sets. We then generated structures after merging data sets belonging to each of these 145 clusters, and we visually inspected each of them to find any recurring patterns. This visual inspection allowed us to determine that all of the reproducible differences could be accounted for using just five of the larger clusters (which we call the green, red, cyan, purple and yellow clusters). In other words, we chose the ‘height’ at which we cut the *KAMO* dendrogram so that five clusters contain the data corresponding to the relevant structures (Fig. 6 ▸). By comparing our results with identically processed data that were modified such that the unit-cell dimensions were either constant or randomized, we observed that the dominant contribution to the clustering correctness derives from differences in the data amplitudes, with differences in the unit-cell parameters playing a lesser role (see supporting information).

The 145 clusters could also be overlaid on the *a* and *c* axis diagram color-coded according to each of the five main clusters (Fig. 4 ▸). All of the data sets of the green and red clusters belong to the *a* = 114–115 Å cluster and the data sets of the cyan, purple and yellow clusters belong to the *a* = 111 Å cluster. If we increase the cut height to 1.5, we obtain sub-master clusters of the two intensities, one containing the green and red clusters and the other containing the cyan, purple and yellow clusters. This means the intensity cluster result has a strong alignment with the unit-cell parameter cluster result.

### 3.3. The five data clusters

**Figure 7:**
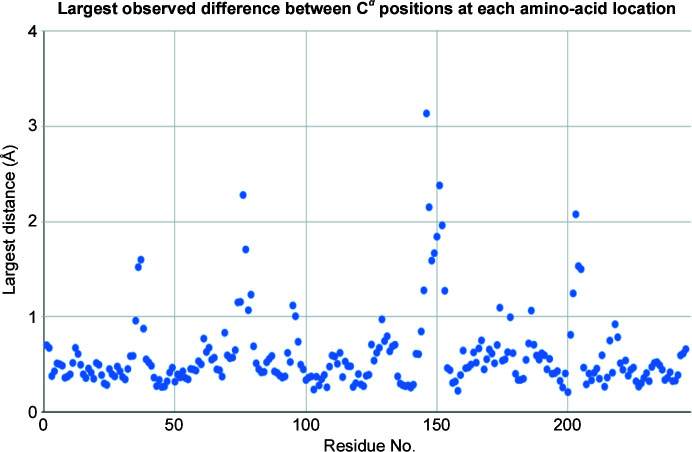
Dot plot of the differences between the C^α^ positions of each residue in the structures. To determine which regions of ChTg were most mobile in our data, we examined the five structures from the five intensity clusters and noted the distances among the C^α^ atoms for each of the 146 amino acids. We plotted the largest value for each amino acid. The data illustrate one extended region with very large variation (between residues 146 and 151, in the vicinity of the missing loop that is a normal cleavage point for α-­chymotrypsin). There are also two shorter regions with smaller variation around Ser75 and Val200.

**Figure 8:**
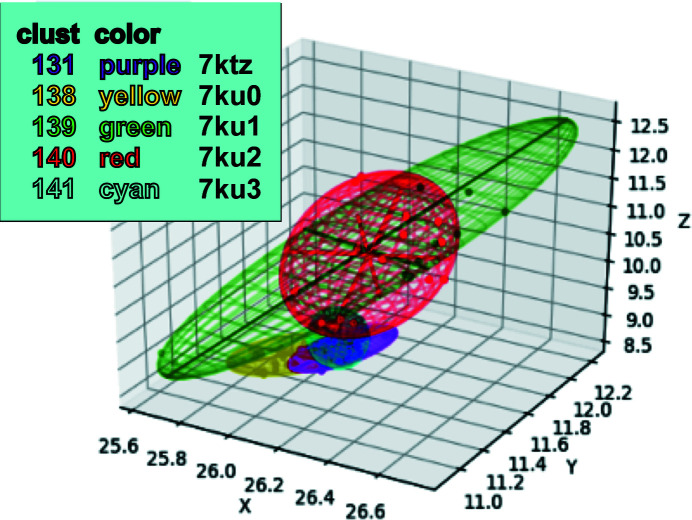
Using ellipsoids to illustrate the variation in the C^α^ coordinate at position 146. We calculated five ellipsoids for each residue position, corresponding to the observed variation in the C^α^ positions at a specific residue for the data in the green, red, cyan, purple and yellow clusters. The lengths of the perpendicular axes were determined using the minimum-volume method (which minimizes the volume of the ellipsoid enclosing the data; see https://github.com/nsls-ii-mx/chymotrypsinogen and https://raw.githubusercontent.com/nsls-ii-mx/chymotrypsinogen/master/ellipsoid.py). This method optimizes the fit of each ellipse to the data, including the major axis in the direction of greatest variation. For example, at C^α^ position 146 (shown here) the green cluster yielded 18 structures with large variation in the [0.2, −0.8, 0.0] direction. The volume of the ellipsoids indicates the overall variation in corresponding C^α^ positions. For example, at position 146 the green and red clusters yielded structures with much larger positional variation than the cyan, purple and yellow clusters.

We generated a dendrogram using Ward’s method for hierarchical clustering with the height cutoff at 1.0 to obtain distinct groups of data sets. To observe the differences between the 145 structures generated using individual data clusters, we calculated the largest differences in physical coordinates at the C^α^ atom of each residue (see Fig. 7 ▸). We observed that the most mobile area, particularly residues 139–145, is near the missing loop from residues 146–152. Note that the distinctive differences between ChTg (the zymogen) and CHT (the enzyme chymotrypsin) are the cleavages at the N-terminus and the gap between Tyr146 and Ala149. The largest differences were observed for residue 146, with average positional differences greater than 3 Å (Fig. 8 ▸).

At each residue position, we plotted ellipsoids to illustrate the variation in the C^α^ coordinates observed in each of the structures corresponding to the five clusters. For example, the ellipsoid for position 146 illustrates that the C^α^ atoms in the green and red clusters have a much greater positional variation (the ellipsoids are bigger) compared with the cyan, purple and yellow clusters. The ellipsoids show the variation of C^α^ coordinates of all structures belonging to each sub-master cluster. The sizes of the ellipsoids show that residue 146 of the structures in the green and the red clusters varies a large amount, while the structures of the cyan, purple and yellow clusters do not change as much.

Table 1 ▸ shows that data sets which belong to the green and red clusters have *a* = *b* unit-cell parameters around 114–115 Å, while data sets in the other clusters have values around 111 Å (Fig. 4 ▸). Table 1 ▸ also reveals that data sets belonging to the cyan, purple and yellow clusters have higher resolution than those belonging to the green and red clusters. The overall resolution of around 2 Å with good structure quality for each of the six structures is indicated by an *R*_work_ and *R*_free_ of about 20%.

**Figure 9:**
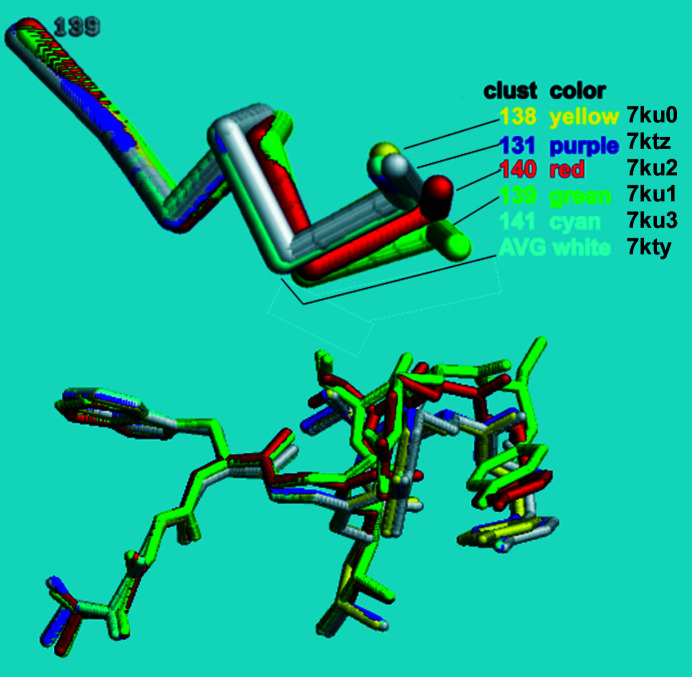
Structural alignment of residues 138–141 displaying the variation in position among the structures representing each cluster. The overall average structure, PDB entry 7kty, which is cluster 145 in the dendrogram, is colored white. PDB entries 7ktz, 7ku0, 7ku1, 7ku2 and 7ku3, which are clusters 131, 138, 139, 140 and 141, are colored purple, yellow, green, red and cyan, respectively. The top half shows the variation in the backbone alone. The bottom half shows the variation including the side chains. Remember that the green and red clusters are structures with *a* = 114–115 Å and all the others have *a* = 111 Å.

When we align the model derived from the average cluster with the five major subclusters using *FATCAT* in rigid mode, all of the residues between 1 and 138 are well aligned, but residues 139–146 increasingly diverge (Fig. 9 ▸).

### 3.4. Detecting dynamic behavior via ligand-binding hotspots

**Figure 10:**
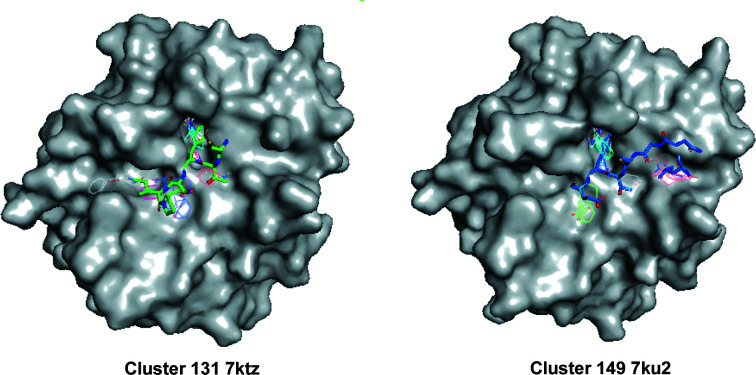
Surface representation of ChTg as calculated by *FTMap* comparing the hotspot areas of clusters 131 (PDB entry 7ktz) and 140 (PDB entry 7ku2). Left: *FTMap* surface representation of the ChTg structure of model 131 (PDB entry 7ktz, the purple cluster with *a* = 111.49 Å) overlapped with a wire-frame rendering of critical parts of a Bowman–Birk plant-based protease-inhibitor complex of chymotrypsinogen (PDB entry 3ru4; Barbosa *et al.*, 2007 ▸). Right: an *FTMap* mapping result of model 140 (PDB entry 7ku2, the red cluster with *a* = 115.57 Å, shown as a Lee–Richards surface) overlapped with a wire-frame rendering of critical parts of the pancreatic Kazal-type inhibitor (PDB entry 1cgi; Hecht *et al.*, 1991 ▸).

*FTMap* shows six binding hotpots for each of the five structures (Fig. 10 ▸; Kozakov *et al.*, 2015 ▸). Among them, we observed the largest differences between the pockets in the structure of the red cluster (PDB entry 7ku2, cluster 140) and the structure of the purple cluster (PDB entry 7ktz, cluster 131). Notably, the pockets with the largest differences overlap with the binding site of the Bowman–Birk protease inhibitor. Since these two structures belong to the two different unit-cell-based clusters, the differences provide strong evidence for the effectiveness of both unit-cell-based and amplitude-based clustering in detecting polymorphs in the case of very small structural changes.

Note that the binding pocket for the Kazal-type inhibitor includes the missing Thr147→Asn150 loop (Tyr146 of ChTg makes two hydrogen bonds to the Kazal-type inhibitor: a direct hydrogen bond to Glu40 and a water-mediated hydrogen bond to Lys43). This would be a characteristic of the active enzyme chymotrypsin. The similarity between the results from data clustering and the results from computer modeling increase our confidence in both methods. We observed additional similarities between the two methodologies, which we are currently investigating.

## 4. Discussion

Although both experimental work (Debrunner & Frauenfelder, 1982 ▸) and theoretical work (McCammon, 1984 ▸) established that dynamic behavior underlies most protein functions, in the early years crystallography was not regarded as an appropriate tool for investigating protein dynamics. An early review of protein crystallography concluded by stating that ‘crystallo­graphic methods are not suitable for the direct study of the dynamics of protein structure and interactions’ (Stryer, 1968 ▸).

However, the presence of diffuse scatter implied that there is dynamic behavior within protein crystals (Caspar *et al.*, 1988 ▸). Crystal structures soon illustrated examples of protein dynamics (Ringe & Petsko, 1985 ▸) that were induced by physical changes such as temperature (Tilton *et al.*, 1992 ▸), pH (Diao, 2003 ▸) and ionic strength (Sanishvili *et al.*, 1994 ▸) and induced by chemical changes by the addition of denaturants (Dunbar *et al.*, 1997 ▸) or ligands (Edwards & Poulos, 1990 ▸).

However, Stryer’s assertion stands to this day in the sense that investigators rarely employ simple tools to identify dynamics from diffraction data, and consequently most crystallographic contributions to dynamics continue to be fortuitous. Specifically, we propose here to provide experimenters with a nearly automatic procedure to suggest insights into the dynamics of proteins by a systematic surveying of diffraction data for the presence of clusters. Once crystallographers are equipped with appropriate tools to identify clusters within aggregates of diffraction data, results indicating dynamic behavior may emerge routinely in many protein crystallo­graphy projects.

A tool to identify dynamic contributions in diffraction data must be as automated as possible, must present results in a way that is easy to interpret and must be sensitive enough to identify small movements. The first of these requirements was simple to accommodate by deploying our software within the existing *KAMO* software package, which we easily integrated into our existing version of the *fast_dp* automated data-analysis pipeline. The experimenter may include this test in the data-reduction pipeline with the flip of a switch, at a reasonably low cost in processing speed. We addressed the second requirement by incorporating visual tools such as systematic color annotation of clusters (Fig. 6 ▸), dot-plot visualization for structure variation (Fig. 7 ▸) and ellipsoid visualization for model variation (Fig. 8 ▸).

The most difficult benchmark was the ability to differentiate clusters where dynamic contributions are small and subtle. We tested our techniques using our data from ChTg, which was not known at the outset to exhibit dynamic behavior. Many of the changes that we identified involved just a few amino acids. By combining the strengths of unit-cell clustering (the ability to operate on thin wedges of data that are often incomplete) and of diffraction-based clustering (a sensitivity to very small structural changes), we believe that our technique will accurately identify relevant clusters of different structures hidden within highly similar data. Our procedure detected different polystates with coordinate differences of less than 3 Å in just two amino acids. In addition, the visualization tools that we created (color-based ellipsoid and scatter plots) allow easy identification of the highly dynamic regions. This provides verification that our clusters are physically meaningful. These tools provide scientists with a simple procedure to screen their data for dynamic behaviors.

High-data-rate crystallography represents a large and growing fraction of all crystallographic data. At synchrotrons, serial crystallography and combinatorial crystallography (for example fragment screening) produce large streams of data from samples that are similar but not identical. One can cluster such data streams automatically, with visual results presented to scientists either to inform their main project or to yield serendipitous information that may expand their thinking about the system in question.

XFEL light sources generate even larger data streams, with individual diffraction images that are derived nearly instantaneously from very small protein crystals. The great reduction in the time and space averaging in XFEL data (compared with synchrotron data) further increases the likelihood of obtaining data from crystals that are in different resolvable polystates. We acknowledge that our software as it stands will not handle the partial data sets produced by the XFEL method. However, eventually the data-processing challenge will be the same: one needs a data-clustering algorithm that is robust enough to work with mixed-quality data, sensitive enough to partition all of the polystates that are present and intuitive enough that investigators can identify useful clusters that represent biologically relevant polystates. Here, we have presented an algorithm that accomplishes these goals.

Our data processing and clustering are automated to reduce the time spent screening and analyzing the molecules. We also perform manual checking to verify that the automated processes achieve reasonable fits to density. However, it is still a challenge if the data contain a lot of noise such as blurred or unindexable spots. This problem may be solved by future research on spot finding and auto-indexing. In addition, we would like to test whether different distance metrics could improve the accuracy of the clustering output and further improve the chances of detecting smaller potentially meaningful changes. We also will test whether the tools can detect polystates well in data sets from other molecules so that we can obtain a comprehensive understanding about the efficiency of our clustering procedure.

## 5. Conclusions

Observing differences in protein structures, even small differences, could be meaningful and important. However, we usually miss changes that are very small since they are very hard to measure. In this paper, we show how one might use the combination of our unit-cell-based and structure-factor-based clustering procedure to detect polystates of molecules. We applied these methods to ChTg data and were able to detect polystates with very small differences among five clusters of data sets. From these clusters, we built molecular structures and verified the differences among them. The combined procedure should help scientists to discover minor changes in molecules that are hardly noticeable from the change of unit-cell parameters only.

We have developed color-based visualization to assist investigators in screening their data for distinct groupings that may represent polystates: dendrograms to show correlations among intensities and scatter plots and ellipsoids to indicate differences in automatically refined structures. The dendrogram shows the members of clusters with custom height cutoffs and the differences among those clusters. The scatter plot quickly shows unit-cell-based clusters and their relations with structure factor-based clusters, and ellipsoids show the variations of the physical coordinates of structures of clusters. Using the color-based plots, one can easily discriminate among groups of data sets. This visualization method is a fast way to screen many data sets and to point out those that are important for further investigation.

## Supplementary Material

PDB reference: chymotrypsinogen, 7kty

PDB reference: 7ktz

PDB reference: 7ku0

PDB reference: 7ku1

PDB reference: 7ku2

PDB reference: 7ku3

Supplementary material. DOI: 10.1107/S2059798321013425/di5049sup1.pdf

## Figures and Tables

**Table 1:** Data collection and processing

PDB code	7kty	7ku1	7ku2	7ku3	7ktz	7ku0
Description	Average	Green	Red	Cyan	Purple	Yellow
Cluster No.	145	139	140	141	131	138
No. of data sets	146	12	16	32	37	49
Wavelength (Å)	0.9201	0.9201	0.9201	0.9201	0.9201	0.9201
Temperature (K)	100	100	100	100	100	100
Detector	EIGER X 9M	EIGER X 9M	EIGER X 9M	EIGER X 9M	EIGER X 9M	EIGER X 9M
Distance (mm)	100–200	100–200	100–200	100–200	100–200	100–200
Rotation (°)	0.2	0.2	0.2	0.2	0.2	0.2
Total range (°)	120	120	120	120	120	120
Space group	*P*4_1_2_1_2	*P*4_1_2_1_2	*P*4_1_2_1_2	*P*4_1_2_1_2	*P*4_1_2_1_2	*P*4_1_2_1_2
*a*, *b* (Å)	114.49	114.49	115.57	111.33	111.49	111.47
*c* (Å)	51.90	51.9	52.92	51.87	52.02	52.36
α, β, γ (°)	90	90	90	90	90	90
Resolution (Å)	2.00	2.39	2.19	2.00	2.00	2.02
No. of reflections	23850	14139	18927	22533	22696	22261
Completeness (%)	99.94	99.80	98.98	99.81	99.93	99.59
〈*I*/σ(*I*)〉	10.91	9.48	9.76	10.66	12.15	10.85
Wilson *B* factor (Å^2^)	53.96	61.87	53.31	33.72	29.15	30.16

**Table 2:** Structure solution and refinement

PDB code	7kty	7ku1	7ku2	7ku3	7ktz	7ku0
Description	Average	Green	Red	Cyan	Purple	Yellow
Final *R*_work_ (%)	19.13	22.08	20.97	18.15	16.18	16.74
Final *R*_free_ (%)	20.19	26.37	23.42	21.01	19.01	19.41
No. of non-H atoms						
Protein	1786	1771	1778	1794	1786	1786
Ligand	15	5	5	15	15	15
Water	99	10	44	195	249	243
Total	1900	1786	1827	2004	2050	2044
R.m.s. deviations						
Bonds (Å)	0.01	0.01	0.01	0.01	0.01	0.01
Angles (°)	0.86	1.00	0.86	0.77	0.80	0.74
Average *B* factors (Å^2^)						
Protein	57.9	67.4	58.7	36.6	28.1	28.8
Ligand	63.0	83.9	75.7	40.7	40.7	34.8
Water	58.7	63.0	56.1	42.3	37.1	36.8
Ramachandran plot (%)						
Favored	97.47	96.20	97.06	97.47	98.31	98.73
Allowed	1.69	2.95	2.52	2.11	1.27	0.84
