# Supplementary material for: A simple technique to classify diffraction data from dynamic proteins according to individual polymorphs
Source: Acta Crystallogr D Struct Biol. 2022 Feb 18;78(Pt 3):268–77. doi: 10.1107/S2059798321013425 (PMC8900820; doi:10.1107/S2059798321013425)

STRUCTURAL
BIOLOGY

Volume 78 (2022)

Supporting information for article:

A simple technique to classify diffraction data from dynamic proteins according to individual polymorphs

Thu Nguyen, Kim L. Phan, Dima Kozakov, Sandra B. Gabelli, Dale F. Kreitler, Lawrence C. Andrews, Jean Jakoncic, Robert M. Sweet, Alexei S. Soares and Herbert J. Bernstein

Supplementary materials: To generate optimal clusters, we combined information from unit cells differences with amplitude differences. To determine what role each of the two sources of information played in the clustering effectiveness, we modified our original data in two ways:

1) We generated a data set that was equal to our original data in all ways but had the a and b unit cell dimensions modified to equal the average (N is the number of data sets):

$$a_{mean} = (1/N) \sum a_{observed}$$
$$b_{mean} = (1/N) \sum b_{observed}$$

2) We generated a data set that was equal to the original data in all ways but had the a and b unit cell dimensions modified to equal a random value equally distributed around the mean value +/- the variance of the mean value (R is a random {-1, 1} and $\sigma(a_{mean})$ is the variance of a_{mean}):

$$a_{random} = a_{mean} + R\ \sigma(a_{mean})$$
$$b_{random} = b_{mean} + R\ \sigma(b_{mean})$$

The KAMO outlier exclusion module removed 8 data sets from the data with correct unit cells, 19 from the data with constant unit cells, and 11 from the data with randomized unit cells. The overall R_{merge} (excluding outliers) was 44.8%, 48.3%, and 45.8% respectively (bar graphs shown in blue at left). We then averaged the individual R_{merge} values for the five clustered data sets ($R_{merge}^{mean} = [1/5] \sum R_{merge}$)(bar graphs shown in orange at right). *This demonstrated a small advantage for the two-factor clustering over single factor clustering* (note different scales shown at left and right).

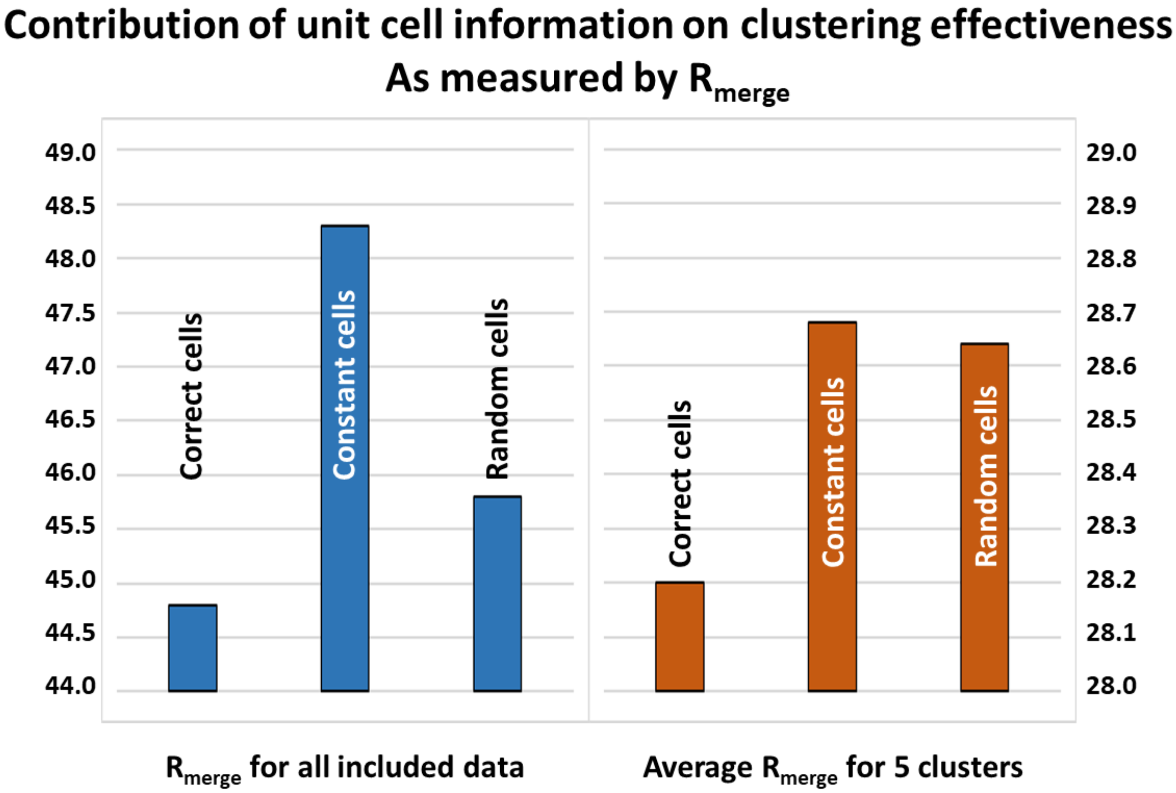

Supplement: Supplementary file 1 [file d-78-00268-sup1.pdf]
